# Achilles' heel of triple negative cancer

**Published:** 2014-12-13

**Authors:** Alberto Ocaña, Juan C. Montero, Atanasio Pandiella

**Affiliations:** Instituto de Biología Molecular y Celular del Cáncer. CSIC-Universidad de Salamanca, Spain

Breast cancer is the most commonly diagnosed tumor in women. While important advances in this field led to progressive increases in survival, in the metastatic setting the disease remains incurable. Because of this, important efforts are being carried out to define the molecular alterations that drive the oncogenic properties of the disease, to therapeutically act on them. In an attempt to better categorize patients to act on their disease more efficiently, several genetic studies have classified breast tumors into different subgroups, according to their gene expression profiles (Curtis et al. Nature 2012; 486:346-52; Perou et al. Nature 2000; 406:747-52). However, gene expression profiling is still not widely used to define treatment of breast cancer, which is mostly guided by anatomopathological criteria (Dawson et al. EMBO J 2013; 32:617-28). Using this approach, breast tumors can be classified into three subgroups: hormone receptor positive (HR+), HER2 positive, or triple negative breast tumors (TNBC). Hormone positive tumors express estrogen and progesterone receptors and are highly sensitive to antihormonal therapies. HER2+ tumors are characterized by overexpression of the transmembrane tyrosine kinase and receive agents against HER2 such as antibodies or small molecule kinase inhibitors. TNBC are characterized by the lack of hormone receptors, and no overexpression of HER2. In contrast to the other two subtypes, no available target is known for patients with TNBC, and their actual treatment is based on classical chemotherapeutics. This fact very much limits successful therapy of TNBC, and for this reason major research efforts aimed at identifying etiopathogenic targets are being carried out (Turner and Reis-Filho, Clin Cancer Res 2013; 19:6380-8).

Genomic studies have revealed deficiencies in DNA repair mechanisms in TNBC (Shah et al. Nature 2012; 486:395-9). As a consequence, agents targeting proteins involved in such mechanisms are under clinical evaluation (Juvekar et al. Cancer Discov 2012; 2:1048-63). How can we identify additional therapeutic vulnerabilities in this type of breast cancer? Phosphokinase profiling has recently been used to analyze the activation status of several oncogenically relevant kinases in TNBC (Montero et al. Oncogene 2014; 33:148-56). Using patient samples as well as representative TNBC cell lines, these studies indicated frequent activation of components of the PI3K/mTOR pathway as well as the ERK1/2 route in this type of tumors. Genetic as well as pharmacological studies indicated an important role of the PI3K/mTOR pathway in the control of the proliferation of TNBC cells both in vitro and in vivo. In contrast, targeting of the ERK1/2 route failed to substantially affect proliferation of TNBC cells. mTOR controls cellular functions through formation of multiprotein complexes, termed mTORC1 and mTORC2. When dissecting the importance of either complex in TNBC, mTORC1 seemed to have a more relevant role than mTORC2 in the control of cellular proliferation. However, when mTOR was neutralized, by either knock down or pharmacological inhibition, the effect on cell proliferation was greater than targeting of mTORC1 or mTORC2 individually. This is therapeutically relevant, since most agents being developed with clinical purposes, i.e. rapamycin and analogues, act mainly on mTORC1. The fact that targeting mTOR was more efficient than exclusively targeting mTORC1, should stimulate an effort to clinically develop mTOR inhibitors instead of mTORC1-directed rapalogs.

The precise mechanisms by which the PI3K/mTOR route is frequently active in TNBC are still unclear. Interestingly, recent massive genomic studies identified certain genetic events which may activate this pathway, including PTEN mutation/deletion in 10%, and PIK3CA mutation in 8% of the tumors. Other genomic alterations which may impinge on this route include EGFR or FGFR amplification (4%) or HER2 mutations (2%) (Shah et al. Nature 2012; 486:395-9; Curtis et al. Nature 2012; 486: 346-52).

Therapeutic inhibition of the PI3K/mTOR pathway in breast cancer is currently an active area of research. Indeed, at this moment several ongoing studies are evaluating PI3K/mTOR inhibitors in TNBC alone or in combination with chemotherapy. However the incorporation of PI3K/mTOR inhibitors into the clinic raises several questions: should PI3K inhibitors be given alone? Can/should they be combined with chemotherapy or targeted agents? How can we select responsive patients?

The preclinical data suggest that combinations with chemotherapy should be a preferred option (Montero et al. 2014). In fact, combination with other targeted agents including PARP inhibitors is under clinical evaluation (Juvekar et al. 2012). Preclinical evidence indicates that PI3K/mTOR targeting in TNBC and other solid tumors causes a cytostatic effect, rather than cytotoxic (Montero et al. 2014). Therefore, full exploitation of the antitumoral effect of the PI3K/mTOR inhibitors probably would require their combination with agents that cause cell death.

A relevant concept is how to identify patients responsive to PI3K/mTOR inhibitors. Some studies have selected patients based on mutations at the PI3KCA gene and/or loss of PTEN. However, recent data suggest that PI3KCA mutations do not predict response to agents against this route (Dillon and Miller. Curr Drug Targets 2014; 15:65-79). This suggests that the best approach to identify a targetable route may be to evaluate its activation status, rather than an analysis of mutational or expression data. In the case of the PI3K/mTOR route, assessment of phosphorylated proteins that can be used as readouts of the activity of the pathway may represent the best method to predict sensitivity to PI3K/mTOR targeting compounds.

In conclusion evaluation of cancer vulnerabilities in triple negative tumors discovered the PI3K/mTOR pathway as a key druggable element. Identification of potential responsive patients and the best partner drug to be combined will be required to optimize the efficacy of these agents in TNBCs.

